# Treatment outcomes and associated factors among tuberculosis patients attending Gurage Zone Public Hospital, Southern Nations, Nationalities, and People's Region, Ethiopia: an institution-based cross-sectional study

**DOI:** 10.3389/fmed.2023.1105911

**Published:** 2023-08-02

**Authors:** Haile Workye Agazhu, Zebene Mekonnen Assefa, Masino Tessu Beshir, Habtam Tadesse, Aregash Sitot Mengstie

**Affiliations:** ^1^Department of Nursing, College of Medicine and Health Science, Wolkite University, Wolkite, Ethiopia; ^2^School of Midwifery Department of Clinical Midwifery, College of Medicine and Health Science, University of Gondar, Gondar, Ethiopia

**Keywords:** tuberculosis, treatment outcome, Gurage Zone, successful treatment outcome, Ethiopia

## Abstract

**Background:**

Tuberculosis remains the most important global health problem. Worldwide, tuberculosis is the cause of a single infectious agent and the ninth leading cause of death, ranking above human immunodeficiency virus. In high-burden settings, one of the mechanisms to control tuberculosis is to identify patients' problems during treatment. Nevertheless, the problem is still a countrywide issue, and there is a shortage of research to show treatment outcomes and associated factors of tuberculosis in Southern Nations, Nationalities, and People's Regions in the Gurage Zone.

**Methods:**

An institution-based, cross-sectional study was conducted to collect data from 347 medical records of tuberculosis patients from 20 July 2016 to 30 July 2021 at Gurage Zone Public Hospitals. The tool includes data about socio-demographic characteristics, as well as tuberculosis-related, and tuberculosis treatment outcome status. Data were analyzed using SPSS version 26, and multivariable logistic regression analyses were conducted to identify significantly associated variables with successful tuberculosis treatment outcomes. The adjusted odds ratio (AOR) with its 95% confidence interval (CI) at a *p*-value of < 0.05 was used to claim statistical association.

**Results:**

In this study, the overall prevalence of successful tuberculosis treatment outcomes was 79.3%. HIV-negative tuberculosis patients (AOR = 4.33; 95% CI: 1.91, 9.79), patients aged < 20 years (AOR = 0.16; 95% CI: 0.04, 0.74), and married participants (AOR = 0.29; 95% CI: 0.10, 0.88) were significantly associated with successful tuberculosis treatment outcomes.

**Conclusion and recommendations:**

The prevalence of successful tuberculosis treatment outcomes was low. HIV-TB co-infection, single marital status, and age >20 years negatively affected the treatment outcomes of tuberculosis, thus more effort and better attention should be given to better outcomes of tuberculosis patients, especially for HIV-TB co-infected participants.

## Introduction

Tuberculosis (TB) is an infectious disease caused by various strains of mycobacteria. Despite the availability of effective drugs, *Mycobacterium tuberculosis* causes a serious health threat among the young, elderly, and immunocompromised people (1). Globally, tuberculosis (TB) continues to be the most prominent cause of death from a single infectious microorganism, and it affects everyone, but people living with HIV infection, health workers, and others in settings with a high risk of transmission of *Mycobacterium tuberculosis* are more prone to this disease (2). In 2018, approximately 10 million people became ill with TB, an estimated 1.5 million died and half a million new people were affected by drug-resistant (DR) TB (3). Globally, TB is the main cause of death and is ranked above HIV/AIDS. A challenge in reducing the burden of TB is undiagnosed or not properly reported TB cases due to the emergence of COVID-19 (4). The 2020 global tuberculosis report indicated that tuberculosis remains the most important health problem, and 43% of new cases of TB occurred in the Southeast Asian Region followed by the African Region (25%) (5). TB is a preventable disease if people have access to healthcare for the diagnosis and are provided the right treatment (6). TB is the leading cause of death for those living with HIV, and the growing HIV epidemic represents a great challenge to TB treatment outcomes. Patients living with TB and HIV are prone to risk for drug-to-drug interaction and immune reconstitution inflammatory syndrome (7). The direct observation therapy (DOT) strategy was launched in 1994, creating the basis for effective TB care and management activities by standardizing the requirements to address epidemic diseases and tackling emerging priorities, such as multi-drug resistant tuberculosis (MDR-TB) and TB/HIV co-infection (8). The global target for TB control is to identify at least 70% of smear-positive patients and to cure at least 85% of the identified patients (4). Successful treatment outcomes of TB may be affected by the incomplete record of the patient's presenting complaints, the time of illness, and physical examination (9). Outcomes of TB tend to be high in populations of low socio-economic status (10). The target of the sustainable development goal (SDGs) is to confirm healthy lives and endorse wellbeing for all at all ages, epidemics of AIDS, TB, malaria, and neglected tropical diseases (11). Community health workers (CHWs) achieved SDG in many low- and middle-income countries although female health extension workers (HEW) in Ethiopia have a unique opportunity to support improved access to health services, including active case finding for TB and treatment (12). A retrospective study conducted at Wolayta Sodo Teaching and Referral Hospital showed that age, type of TB, HIV status, and residence are factors that affect TB treatment outcomes (13). A study conducted in the public hospital of Harar town revealed that TB outcomes are affected by sex, age, pretreatment weight, HIV status, and TB patient category (14). In a study conducted in Bale Zone, Southeast Ethiopia, the prevalence of successful TB outcomes is 87.8, and age, type of TB, treatment adherence support center, and year of treatment for patients are factors that affect TB outcomes (15). The percentage of patients treated successfully is a key indicator for monitoring and evaluating the effectiveness of the TB DOTs program to assess the level of quality of care and to imply possible directions for improvement. Assessing TB treatment outcomes and factors that affect successful TB treatment is important for policymakers and healthcare providers in forecasting interventions to overcome the barriers and improve patient treatment response. Hence, this study aims to assess TB treatment outcomes and associated factors among patients who had received treatments for TB in the public hospital of Gurage Zone, Southwest Ethiopia, from 20 July 2016 to 30 July 2021.

## Methodology

### Study area and period

The study was conducted in the public hospital of Gurage Zone, SNNPR, Ethiopia. It is located at a distance of 158 Km from Addis Ababa. In the Gurage Zone, there are six public hospitals that provide clinical services including emergency, gynecology/obstetrics, surgery, pediatrics, ART, TB clinic, and dental clinic. This town has a total population of 1,34,683, of whom 67,130 are men and 67,553 are women. A 5-year institution-based cross-sectional analysis of TB treatment outcomes was conducted among TB patients registered from 20 July 2016 to 30 July 2021.

### Study population and source of population

All TB patients who were registered from 20 July 2016 to 30 July 2021 in public hospitals of the Gurage Zone were the source of the population. In the selected three public hospitals, all TB patients who were registered in the study period were the study population, and patients with incomplete data were excluded from the study.

### Sample size calculations and sampling procedures

A single population formula was used to calculate the sample size, i.e., *n* = z^2^ p (1 – p)/d^2^, where z is the normal standard deviation set at 1.96, the confidence level is specified at 95%, and the margin of error (d) is 5%, the non-response rate is 10%, and the prevalence of successful TB outcome (p) is 70.7%, as mentioned in a previous study conducted from January to June 2011 in Gambella Regional hospital (16). The calculated sample size for this study was 319, and by adding 10% (non-response rate), it gives 350. By using the lottery method, we selected three hospitals from six hospitals. For each health facility based on the annual report, proportional allocation was done by using the number of TB patients registered from 2016 to 2021. After that, the systematic sampling procedure was used to select TB patients registered in the study period. The sampling interval (K) was obtained by dividing the estimated total number of annual registered TB patients 985 by the number of sample sizes 350 (n) at each data collection site. By using the lottery method, the first case was randomly selected. Until the required sample size is reached, every third record was selected.

### Data collection procedure and measurement tool

A pre-test was conducted at Wolkite specialized hospital by using 5% of the sample to check the wording. Data were extracted from the TB registration books of selected hospitals from 20 July 2016 to 30 July 2021, by nurses with B.Sc. qualifications using a structured data sheet that was developed to capture data from the TB registration book. The data sheet was prepared by reviewing the TB registration book of those hospitals containing basic information such as age, sex, address, category of TB, HIV status, acid-fast bacilli (AFB) result at the baseline, monthly treatment regimen, and treatment outcomes. Outcome definitions are classified as successful (cured or treatment completed) and unsuccessful (death, loss to follow-up, failure, or transferred-out) outcomes. The data were collected by five BSc nursing staff and supervised by one MSc nursing staff and a principal investigator. For data collectors and the supervisor, 2 days of training were given.

### Data processing and analysis

The collected data were coded, checked, and entered into the Epi-data version 4.6 statistical software, they were then imported to SPSS version 26 for analysis. The result of the univariable analysis (descriptive results) was presented as frequencies and percentages. The chi-square assumption was checked before bivariable analysis. The bivariable analysis was carried out to check the association of each independent variable with the outcome variable. Variables that have an association with the outcome variable at a *p-*value of ≤ 0.2 were selected as candidates for multivariable analysis. The multivariable analysis was performed in the logistic regression. Hosmer and Lemeshow tests were conducted to test the goodness of fit model. Odds ratios (OR) with 95% CI were used to show the strength and direction of the associations. Finally, a variable with a *p*-value of < 0.05 was considered to be statically significant.

### Ethical approval

Before beginning data collection, an ethical letter was obtained from the Department of Nursing at the Wolkite University College of Medicine and Health. Upon submitting the letter to the hospital administration and after getting consent from the above bodies, the TB registration book was accessed.

## Results

### Socio-demographic characteristics

Approximately 347 registered tuberculosis cases were included, giving a 99.1% response rate. Nearly half of the cases (168; 48.4%) were within the age group of 21–40 years and among the participants, 167 (51.1%) were women (Table 1).

**Table 1:** Socio-demographic characteristics of tuberculosis patients in Gurage Zone Public Hospital SNNPR, Ethiopia, from July 2016 to January 2021 (*n* = 347).

**Variables**	**Category**	**Frequency**	**Percent (%)**
Age	≤ 20 years	56	16.1
	21–40 years	168	48.4
	40–60 years	90	25.9
	>60 years	33	9.5
Sex	Female	167	48.1
	Male	180	51.9
Residence	Urban	292	84.1
	Rural	55	15.9
Marital status	Single	137	39.5
	Married	173	49.9
	Divorced	12	3.5
	Widowed	25	7.2

### Clinical characteristics

More than one-third of the participants (34%) presented with extrapulmonary TB. Most of the attendants (88.2%) were newly treated, and approximately 11% of the patients were HIV-positive of whom 94.4% were on ART. Among the participants, 94.5% of patients received treatment for 6 months (Table 2).

**Table 2:** Clinical characteristics of tuberculosis patients in Gurage Zone Public Hospital SNNPR, Ethiopia, from July 2016 to January 2021 (*n* = 347).

**Variable**	**Category**	**Frequency**	**Percent**
Types of TB by location	PTB	229	66
	Extra PTB	118	34
Types of TB by AFB smear	Smear positive	145	41.8
	Smear negative	202	58.2
Patient category	Newly treated	306	88.2
	Relapse after treatment	21	6.1
	Return after default	15	4.3
	Failure after treatment	5	1.4
HIV comorbidity	Yes	38	11
	No	309	89
If yes, started ART	Yes	34	94.4
	No	4	5.6
Previous drug use	New	280	80.7
	First line	55	15.9
	Second line	12	3.5
Duration of treatment	6 months	328	94.5
	8 months	7	2.0
	18 months	12	3.5
Sputum result	Positive	207	59.7
	Negative	140	40.3
Weight at start	< 33 kg	44	12.7
	33–54 kg	271	78.1
	55–70 kg	32	9.2
Weight in the continuation phase	< 33 kg	13	3.7
	33–54 kg	145	41.8
	55–70 kg	185	53.3
	>70 kg	4	1.2

### Tuberculosis treatment outcome

**Figure 1:**
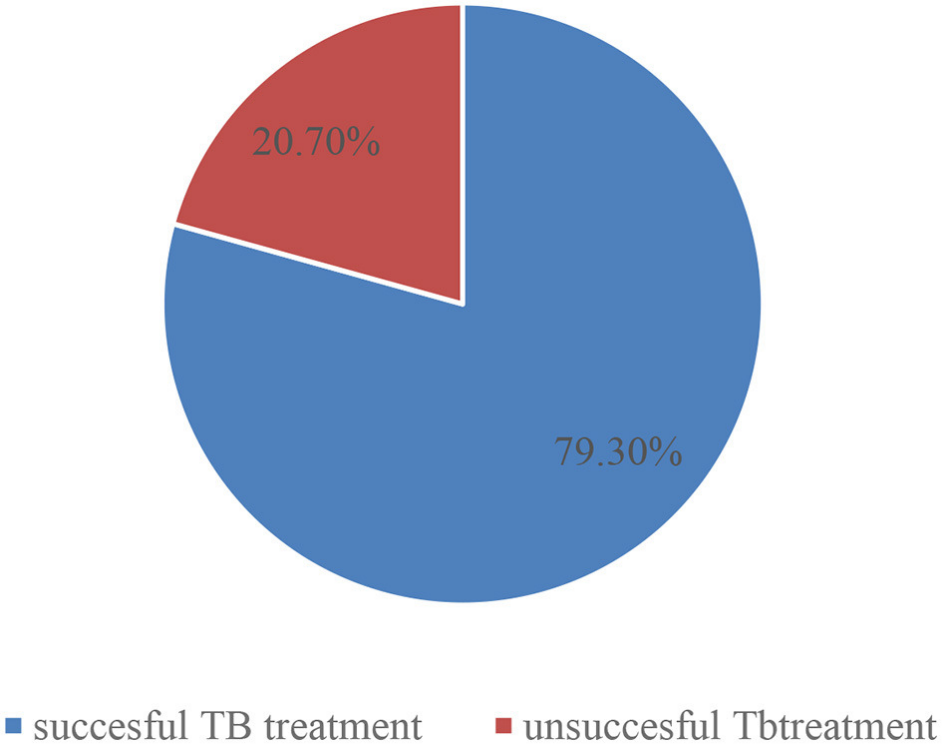
Treatment outcomes of tuberculosis among tuberculosis patients registered in Gurage Zone Public Hospital SNNPR, Ethiopia, July 2016 to January 2021 (*n* = 347).

Of the total 347 registered cases, 275 (79.3%) had a successful treatment outcome. Among those patients who received treatment, 24.5% were cured and 54.8% had completed their treatment regimen. Approximately 20.7% had unsuccessful outcomes; among those, 13.8% of cases had treatment failure, 4.6% of cases had died, and 2.3% of cases had defaulted (Figure 1).

### Factors associated with tuberculosis outcome

In binary logistic regression, age, marital status, residence, and HIV status were associated factors, but in the multivariable logistic regression, age, marital status, and HIV status were significantly associated with successful treatment outcomes. The odds of having successful TB treatment outcomes were 84% lower among patients who were aged < 20 years compared to those who were aged above 60 years (AOR = 0.16; 95% CI: 0.04, 0.74). TB patients with husbands were 71% lower in having successful TB treatment outcomes compared to widowed patients. HIV-negative tuberculosis patients were 4.3 times more likely to have successful treatment outcomes than HIV-positive tuberculosis patients (AOR = 4.33; 95% CI:1.91, 9.79) (Table 3).

**Table 3:** Factors associated with treatment outcome among tuberculosis patients registered in Gurage Zone Public Hospital SNNPR, Ethiopia, from July 2016 to January 2021 (*n* = 347).

**Variable**	**Category**	**Successful outcome**	**Unsuccessful outcome**	**Crude odds ratio with 95% CI**	**AOR with 95%CI**
Age	≤ 20 years	50	6	0.069 (0.023–0.207)	0.16 (0.04–0.74)^*^
	21–40 years	147	21	0.082 (0.035–0.190)	0.17 (0.06–0.50)^*^
	41–60 years	66	24	0.208 (0.089–0.486)	0.30 (0.12–0.83)^*^
	>60 years	12	21	**1.00**	**1.00**
Marital status	Married	136	37	0.13 (0.05–0.32)	0.29 (0.10–0.88)^*^
	Single	121	16	0.06 (0.02–0.17)	0.30 (0.07–1.65)
	Divorced	10	2	0.09 (0.02–0.53)	0.23 (0.03–1.68)
	Widowed	8	17	**1.00**	**1.00**
Residence	Rural	40	15	**1.00**	**1.00**
	Urban	235	57	0.65 (0.33–1.25)	0.77 (0.36–1.65)
HIV comorbidity	Positive	18	20	**1.00**	**1.00**
	Negative	257	72	0.18 (0.90–0.37)	4.33 (1.91–9.79)^**^

1, Reference category; ^*^p > 0.01 and p ≤ 0.05; ^**^p ≤ 0.01.

## Discussion

In this study, the prevalence of successful tuberculosis treatment outcomes among tuberculosis patients registered in Gurage Zone public hospital SNNPR, Ethiopia, from 20 July 2016 to 30 July 2021 and its possible association with various variables were assessed. The prevalence of successful tuberculosis treatment outcomes was 79.3%, which is nearly similar to the findings of the study conducted at Gondar (77.3%) (17), Wolayta Sodo (82.5%) (13), Woldia (80.7%) (18), and Cameron (78.6%) (19), but higher than the study conducted at Gambella 70.7% (16), Ethiopian University Hospital (60.1%) (7), Southwest Ethiopia (71.4%) (20), Western Ethiopia (60.7%) (21), and Ekiti State University (68.1%) (22). This variation may be due to differences in the study period and different sample sizes, and the possible explanation might be due to the increased availability to access health services. However, the prevalence of this study was lower than the studies conducted at Jimma University (88.5%) (23), Bale Zone (87.8 %) (15), Harar Town (92.5%) (14), and Addis Ababa (94.6%) (24). This discrepancy may be due to the difference in socioeconomic and accessibility of health institutions because better accessibility to information and accessibility to health institutions in the case of the distance of the health institutions from the participant's home and accessibility of transport is important for better cooperation in the management of TB and leads to better outcomes of TB treatment but the study area has a challenge for it. Additionally, the variation might be due to the difference in study areas, and this study was performed in both urban and rural, but the study conducted in Addis Ababa was only for urban patients. Most of the time, urban communities take the BCG vaccine to protect children rather than to interrupt transmission among adults and knew about TB transmission, high information about their treatment, and appropriate health-seeking behavior.

As reported by some studies (14, 15, 18), younger patients had a high chance of successful TB treatment outcomes than older patients. However, the odds of having a successful TB treatment outcome were 84% lower among patients of a younger age. This difference may be due to variations in the study area. In this study area, the younger population was more exposed to alcohol drinking and cigarette smoking in the area of study, and also the sociocultural khat use pattern was considered normative khat use, thus they are immunosuppressed, and they have less successful TB treatment outcomes. Married TB patients were 71% lower in having successful TB treatment outcomes, the reason might be that married TB patients who have sexual intercourse may produce a high amount of ATP. In addition to this, married TB patients have close contact with their spouses, and this might lead to a high chance of re-infection with tuberculosis. In a systematic review and meta-analysis study conducted in Ethiopia, unmarried individuals were more likely to have a delayed diagnosis of tuberculosis (25). HIV-negative tuberculosis patients were 4.3 times more likely to have successful treatment outcomes than HIV-positive tuberculosis patients. This finding is supported by a study conducted in Harar (14), Southwest Ethiopia (20), Addis Ababa (24), and Woldia (18). The possible reason may be that HIV-positive TB patients have weak immunity compared to HIV-negative TB patients, which leads to increased active TB infection, re-infection, or reactivation. It also increases the risk of TB progression from latent TB to active TB disease. The other justification is that HIV-positive TB patients did not take medication properly due to the fear of side effects and drug interaction.

### Limitations of the study

This study is a document review, and it may not be complete or be written in an objective fashion, so a critical stance has been adopted and it should not be assumed that the information contained within them is precise or unbiased.

## Conclusion and recommendation

In this study, the prevalence of successful TB treatment outcomes was low (79.3 %), which is below the defined standard of an 85% threshold. Age, marital status, and HIV status were significantly associated with successful treatment outcomes. HIV remains a key risk factor for the development of active TB infection. HIV-positive patients should get proper screening to prevent the occurrence of TB and proper management to reduce unsuccessful TB treatment in patients with HIV-TB co-infection. In addition, further prospective studies are needed to identify other potential sociodemographic and behavioral factors that could affect the treatment outcomes of TB patients.

## Data availability statement

The original contributions presented in the study are included in the article/supplementary material, further inquiries can be directed to the corresponding author.

## Author contributions

HA and AM performed the study design, analysis, and report writing and drafted the manuscript. MB, HT, and ZA were involved in reviewing the study design, analysis, and manuscript. All authors read and approved the final manuscript.
